# Chronodisruption and Loss of Melatonin Rhythm, Associated with Alterations in Daily Motor Activity and Mitochondrial Dynamics in Parkinsonian Zebrafish, Are Corrected by Melatonin Treatment

**DOI:** 10.3390/antiox12040954

**Published:** 2023-04-18

**Authors:** Paula Aranda-Martínez, José Fernández-Martínez, Yolanda Ramírez-Casas, César Rodríguez-Santana, Iryna Rusanova, Germaine Escames, Darío Acuña-Castroviejo

**Affiliations:** 1 Centro de Investigación Biomédica, Facultad de Medicina, Departamento de Fisiología, Instituto de Biotecnología, Parque Tecnológico de Ciencias de la Salud, Universidad de Granada, 18016 Granada, Spain; 2 Centro de Investigación Biomédica en Red Fragilidad y Envejecimiento Saludable (CIBERFES), Ibs.Granada, Hospital Universitario San Cecilio, 18016 Granada, Spain; 3 UGC de Laboratorios Clínicos, Hospital Universitario San Cecilio, 18016 Granada, Spain

**Keywords:** zebrafish, Parkinson disease, clock genes, chronodisruption, mitochondria, melatonin

## Abstract

Beyond sleep/wake, clock genes regulate the daily rhythms of melatonin production, motor activity, innate immunity, and mitochondrial dynamics, among others. All these rhythms are affected in Parkinson’s disease (PD), suggesting that chronodisruption may be an early stage of the disease. The aim of this study was to evaluate the connection between clock genes and these rhythms in PD, and whether melatonin administration reestablished the normal clock function. Parkinsonism was induced with 600 μM MPTP (N-methyl-4-phenyl-1,2,3,6-tetrahydropyridine) in 24–120 h post fertilization (hpf) zebrafish embryos and melatonin was administered at a dose of 1 μM. Day–night melatonin rhythm disappeared in MPTP-treated embryos, which showed an advance in the activity phase in parallel with changes in the rhythm of clock genes. An alteration in the fission-to-fusion mitochondrial dynamics was also detected in parkinsonian embryos, increasing the former and leading to apoptosis. Melatonin administration to MPTP-treated embryos fully restored the circadian system, including the rhythms of clock genes, motor activity, melatonin rhythm, and mitochondrial dynamics, and decreasing apoptosis. Because clock-controlled rhythms such as sleep/wake alterations are early events in PD, the data here reported may point to chronodisruption as one initial pathophysiological event of the disease.

## 1. Introduction

Parkinson’s disease (PD) is the second most common neurodegenerative disease. Among other symptoms, PD patients commonly report sleep disturbances [1], which may appear years or decades before the appearance of motor symptoms, when the disease is diagnosed [2]. The sleep–wake cycle is the most evident circadian rhythm, but other rhythms, including that of catecholamines [3], melatonin production [4], and motor activity [5], are also altered in PD. Because circadian rhythms are controlled by the so-called clock genes that make up the biological clock, the connection between chronodisruption, i.e., the failure of the biological clock, and the pathophysiology of PD, has been suggested.

From a molecular point of view, the biological clock in mammals includes *clock* and *bmal1* genes and the corresponding proteins Clock and Bmal1, which dimerize and activate gene transcription by binding to E-box elements in the promoters of different genes. Some of these genes are *per* and *cry*, giving the Per and Cry proteins, which form homo and heterodimers that enter the nucleus and bind to Clock/Bmal1 dimers, inhibiting their transcriptional activity, and closing the loop of transcriptional and translation activities that underlie the operative biological clock. Besides this primary loop, *rorα* and *rev-erbα* are also under the transcriptional control of Clock/Bmal1, giving the corresponding proteins Rorα and Rev-erbα, which bind to *rorα* response elements (ROREs) in the promoter of *bmal1* to activate and to inhibit its transcription, respectively, thus affecting the Clock/Bmal1 activity [6,7]. Chrono, another clock protein, modulates the clock loop by inhibiting the transcriptional activity of Bmal1 [8,9].

Unlike other vertebrates, zebrafish (*Danio rerio*) present several copies of each clock gene due to a duplication of the genome. From the *bmal* gene, it contains *bmal1a*, *bmal1b*, and *bmal2*, and from *clock*: *clock1a*, *clock1b*, and *clock2*. All copies of both genes exhibit the same circadian rhythm and dimerize in different combinations. Zebrafish have four genes for *per*: *per1a*, *per1b*, *per2*, and *per3* and six copies for *cry*: *cry1a*, *cry1b*, *cry2*, *cry3a*, *cry3b*, *cry4*, and *cry5*. Only Cry1a, Cry1b, Cry3a, and Cry3b repress Bmal1/Clock. *Per2* and *cry1a* are directly regulated by light [6].

In mammals, the biological clock is in the suprachiasmatic nuclei of the hypothalamus, and it is controlled by the photoperiod via the information provided by the optic nerve through the suprachiasmatic nucleus, giving a period of oscillation of 24 h [10]. In zebrafish, the clock is contained in the pineal gland, which acts as a direct photoreceptor to control the 24 h oscillatory activity of the clock genes [11,12]. Moreover, and in line with other vertebrates, the clock controls melatonin production, which increases at night and decreases during the day [11]. Pineal melatonin is released into the circulation and reaches all the cells of the body, constituting the chronobiotic signal that synchronizes rhythms including circadian behavior [13,14]. Melatonin also improves mitochondrial function, enhancing OXPHOS and increasing ATP production [15].

Previous reports showed that melatonin exerts neuroprotective properties in parkinsonism models of vertebrates including zebrafish [16,17]. Here, we considered it worthwhile to examine the status of the clock genes’ rhythms during the development of parkinsonism in zebrafish, and whether a process of chronodisruption may be involved in the initial pathophysiological events.

## 2. Materials and Methods

### 2.1. Fish Maintenance

Adult zebrafish (*Danio rerio*) of the AB line were provided by ZFBiolabs S.L (Madrid, Spain) and were maintained in a recirculation aquaculture system (Aquaneering Incorporated, Barcelona, Spain) in the University of Granada’s facility at a water temperature of 28.5 ± 1 °C and under a photoperiod of 14:10 h (lights on at 08:00 h). The maintenance, feeding, breeding, anesthesia, and sacrifice of the fish were carried out according to established protocols [18]. Adult zebrafish were used as breeding stocks and embryos were maintained in E3 medium. All experiments were performed in accordance with the National Institutes of Health Guide for the Care and Use of Laboratory Animals, the European Convention for the Protection of Vertebrate Animals used for Experimental and Other Scientific Purposes (CETS # 123), and the Spanish law for animal experimentation (R.D. 53/2013). The protocol was authorized by the Andalusian’s Ethical Committee (#29/05/2020/068).

### 2.2. Treatments

**Figure 1:**
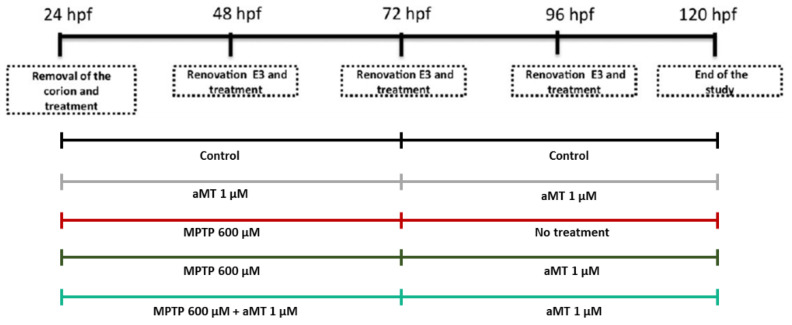
Schedule of the experimental conditions.

Embryos at 24 hpf (hours postfertilization) were manually dechorionated and distributed in a 24-well plate containing six embryos per well and with 1 mL of E3 medium, and the embryos were maintained with or without the treatment up to 120 hpf. The experiments were performed in triplicate, and the following groups were assigned: (1) control group, with no treatment; (2) MPTP group, treated with MPTP (N-methyl-4-phenyl-1,2,3,6-tetrahydropyridine) (Sigma-Aldrich, Madrid, Spain) freshly prepared in E3 medium at a final concentration of 600 μm in the well; (3) Melatonin group, treated with melatonin (Fagrón Ibérica, Barcelona, Spain) dissolved in 0,1% dimethylsulfoxide (DMSO) and used at a final concentration of 1 μm; (4) MPTP+Melatonin curative group, in which MPTP was maintained from 24 hpf to 72 hpf and melatonin was administered from 72 hpf to 120 hpf; and (5) MPTP+Melatonin preventive group, in which MPTP was maintained from 24 hpf to 120 hpf, and melatonin was administered together with MPTP at 24 hpf and was maintained up to 120 hpf. Each day, half of the treatment or E3 medium was renewed. At 120 hpf, the embryos were sacrificed using tricaine (Figure 1).

### 2.3. Activity Rhythm

Zebrafish embryos at 120 hpf were raised in 48-well plate containing 1 mL of E3 medium with or without treatment. Total distance travelled (mm) of each fish was recorded by ZebraBox (Viewpoint, Lyon, France) in tracking live mode. The experiment was carried out during 24 h, with a cycle of 14 h of light and 10 h of darkness. The detection threshold was 25, and 0 mm/s was used for the inactivity threshold. Activity was recorded every 30 min and the data were collected with Zebralab software version 3.22.3.9. 

### 2.4. Determination of Melatonin Concentration

The fish were collected at 120 hpf at the end of the day and at the end of the night for subsequent analysis of melatonin concentration by UHPLC–MS/MS (Ultra-high performance liquid chromatography tandem mass spectrometry) spectrometry. Melatonin was extracted in chloroform, evaporated, and reconstituted in mobile phase. Then, 25 µL were injected onto the UltiMate 3000 UHPLC system (ThermoFisher Scientific, Madrid, Spain) equipped with a Hypersil GOLD 100 mm × 2.1 mm, and 1.9 μm C18 reverse phase column (ThermoFisher Scientific, Madrid, Spain). The mobile phase used consisted of water with 0.1% (*v*/*v*) formic acid (A) and acetonitrile containing 0.1% (*v*/*v*) formic acid (B). A linear gradient was used over 7 min from 5% to 98% B and held at 98% acetonitrile for 2.1 min, followed by 0.9 min equilibration to 5% B, resulting in a total analysis time of 10 min. The mobile phase flow rate was 0.4 mL/min and the autosampler and column oven temperatures were 10 °C and 45 °C, respectively. Then, melatonin was detected by an Orbitrap Q-Exactive Focus mass spectrometer (ThermoFisher Scientific, Madrid, Spain), controlled by Xcalibur software *v.* 4.1.31.9 (ThermoFisher Scientific, Madrid, Spain) in the positive electrospray ionization (ESI) mode using a selective ion monitoring scan (SIM). Protonated melatonin (m/z 233.12845) was detected at a retention time of 3.98 min. 

### 2.5. Gene Expression Analysis

RNAwas extracted with RNeasy Plus Mini kit (Qiagen, Hilden, Germany) and electrophoresed in 1.5% agarose to check RNA integrity. RNA was quantified by optical density at 260/280 nm, and it was used to generate cDNA with the qScript cDNA Synthesis Kit (Quanta bio, Beverly, MA, USA). Amplification was performed with PerfeCTa SYBR Green FastMix Low ROX kit (Quanta bio, Beverly, MA, USA) in the Stratagene Mx3005P (Agilent Technologies, Santa Clara, Madrid, Spain). Forward and reverse primers for each gene are depicted in Table 1.

### 2.6. Circadian Rhythm Analysis of the Clock Genes Expression

Rhythmicity analysis of clock genes expression was performed with a single-component cosinor model with the Time Series Analysis-Seriel Cosinor 6.3 Lab View software (TSASC 6.3; Expert Soft Technologies Inc., BioMedical Computing and Applied Statistics Laboratory, Esvres, France). We assumed a period of 24h and the rhythm detection was considered statistically significant at *p* < 0.05 with 95% confidence limits. The Cosinor program calculated the MESOR (midline estimating statistic of rhythm, which reflects the rhythm-adjusted mean), the amplitude (difference between the mesor and the maximum or minimum value of the best-fitting cosine curve), and the acrophase (time period at which the peak of cosine curve occurs).

### 2.7. Western Blot Analysis

Zebrafish embryos were homogenized in 50 mm Tris–HCl, 0.15 m NaCl, 1% Triton X-100, 1 mm DTT, (1,4-ditiotreitol) pH 7.6 with a protease inhibitor cocktail (Thermo Scientific, Madrid, Spain), sonicated, and centrifugated at 1000× *g* for 5 min at 4 °C. Proteins were quantified by Bradford assay and denatured at 99 °C for 5 min. Then, 40 μg of protein of the sample was loaded in 10 or 12% SDS-PAGE (sodium dodecyl sulfate polyacrylamide gel electrophoresis) gel and the electrophoresis was carried out using the mini-PROTEAN Tetra Cell electrophoresis system (Bio-Rad, Madrid, Spain). The transfer of the proteins to the PVDF (polyvinylidene difluoride) membrane was carried out in a mini Trans-blot Cell (Bio-Rad, Madrid, Spain) and protein–antibody interactions were detected with peroxidase-conjugated horse anti-mouse or anti-rabbit IgG (immunoglobulin G) antibodies using Clarity™ Western ECL Substrate (Bio-Rad, Madrid, Spain). Bands were detected with the ChemiDoc MP Imaging System (Bio-Rad, Madrid, Spain) and quantified by Image Lab Software (Bio-Rad, Madrid, Spain). The primary antibodies were anti-Dnm1l/Drp1 (PA-43802, Thermo Fisher scientific, Madrid, Spain), anti-Mfn2 (TA344104, OriGene, Herford, Germany), anti-Opa1 (CPA3687, Cohesion biosciences, Madrid, Spain), anti-Bax (sc-7480, Santa Cruz Biotechnology, Heidelberg, Germany), anti-P53 (sc-6243, Santa Cruz Biotechnology, Heidelberg, Germany), anti-Lc3 (NB100-2220, Novus Biologicals, Madrid, Spain), and anti-Rpl13a (ab96074, Abcam, Cambridge, UK) as a standard loading control. 

### 2.8. Statistical Analysis

Statistical analyses were carried out using the GraphPad Prism *v.* 8.0.1 software (GraphPad, Software, Inc., La Jolla, CA, USA). Data are expressed as the mean ± S.E.M. Unpaired *t* test and One-way or Two-way ANOVA with a Tukey’s post hoc test were used to compare the differences between the experimental groups. A *p*-value of 0.05 was considered to be statistically significant. 

## 3. Results

### 3.1. MPTP Blunts the Day/Night Melatonin Rhythm, and It Is Restored by Melatonin Treatment

**Figure 2:**
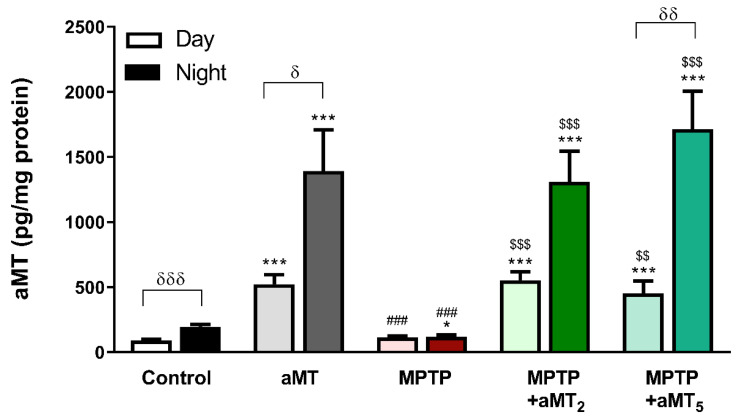
Endogenous levels of melatonin in zebrafish embryos during the day (light color) and during the night (dark color). Control, untreated embryos; aMT, control embryos treated with 1 μM melatonin; MPTP, embryos incubated with 600 μM MPTP; MPTP+MT_2_, embryos treated with 600 μM MPTP from 24 hpf to 72 hpf plus 1 μM melatonin from 72 hpf to 120 hpf; MPTP+aMT_5_, embryos treated with 600 μM MPTP from 24 hpf to 72 hpf plus 1 μM from 24 hpf to 120 hpf. Data are presented as mean ± SEM. * *p* < 0.05 vs. control; *** *p* < 0.001 vs. control, ### *p* < 0.001 vs. aMT; $$ *p* < 0.01 vs. MPTP; $$$ *p* < 0.001 vs. MPTP; δ *p* < 0.05 vs. day; δδ *p* < 0.01 vs. day; δδδ *p* < 0.001 vs. day. Unpaired *t* test.

The daily content of melatonin in the zebrafish embryos was analyzed by mass spectrometry (Figure 2). The control group had a typical rhythm of endogenous melatonin production, significantly increasing at night. Zebrafish primed with 1 μM melatonin (aMT) displayed a similar day/night rhythm, although the concentrations were much higher with respect to the controls. The day/night content of melatonin was significantly reduced in embryos treated with 600 μM MPTP compared with the controls, also blunting the rhythm of the indoleamine in a similar way as in PD patients [19]. The preventive administration of melatonin for two days (MPTP+aMT_2_) fully counteracted the inhibition of the endogenous melatonin production by MPTP, reestablishing the day/night rhythm at the level of the aMT group. Lastly, the curative treatment with melatonin (MPTP+aMT_5_) also significantly increased its levels and totally counteracted the effects of MPTP. 

### 3.2. Melatonin Restores the Motor Activity Rhythm Disrupted by MPTP 

**Figure 3:**
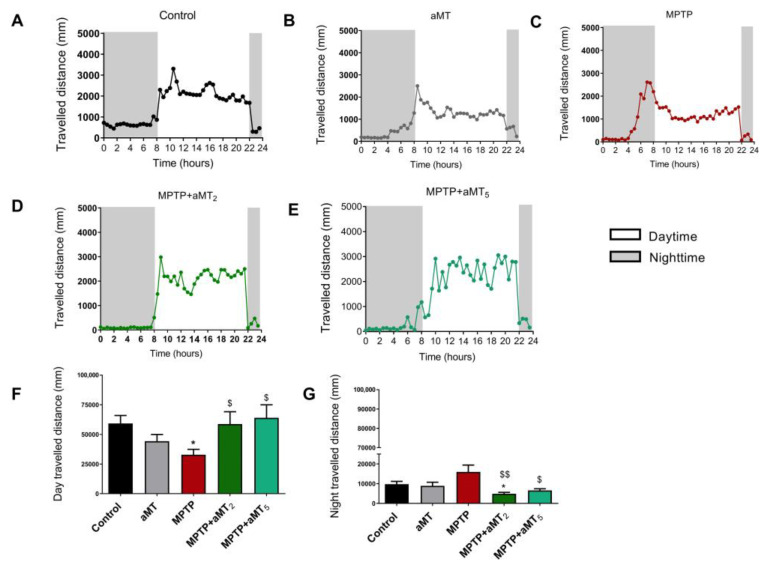
Total distance traveled during 24 h by zebrafish embryos. The light and dark conditions (shaded part) are 14:10 h, respectively. (**A**) control rhythm; (**B**) aMT group; (**C**) MPTP group; (**D**) MPTP+aMT_2_ group; (**E**) MPTP+aMT_5_ group; (**F**) total day travelled distance; and (**G**) total night travelled distance. Data are presented as mean ± SEM. * *p* < 0.05 vs. control; $ *p* < 0.05 vs. MPTP; $$ *p* < 0.01 vs. MPTP. Unpaired *t* test.

Because motor skills are affected in PD [20], we analyzed here whether the activity was affected by MPTP in zebrafish embryos. The control and aMT groups displayed a typical day/night rhythm of activity, with low activity at night (shaded part of the graph) (Figure 3A,B, respectively). Melatonin treatment, however, reduced the activity of the control embryos during the day (Figure 3F). MPTP produced an advance of the activity towards the sleep phase and before the lights came on (Figure 3C). Consequently, the distance travelled by the embryos, although decreasing during the day, increased at night in MPTP compared with the control and aMT groups (Figure 3F,G, respectively). Both preventive (aMT_2_) and curative (aMT_5_) treatments with melatonin restored the rhythm of activity to the control values (Figure 3D,E, respectively). These effects are reflected in the recovery of the night and day travelled distances to the control values (Figure 3F,G).

### 3.3. MPTP Changes in Circadian Rhythms of Clock Genes Are Normalized after Melatonin Treatment

The alterations in the day/night melatonin levels and in the motor activity rhythm in parkinsonian zebrafish embryos may underlie the existence of a process of chronodisruption. To get more insights into this phenomenon, we analyzed the expression of the clock genes at different time points during a 24 h period in the same experimental groups of zebrafish embryos. The statistical data of the cosinor analysis of clock genes rhythms, including the percentage of the rhythms, and their significance, are depicted in Supplementary Table S1. 

**Figure 4:**
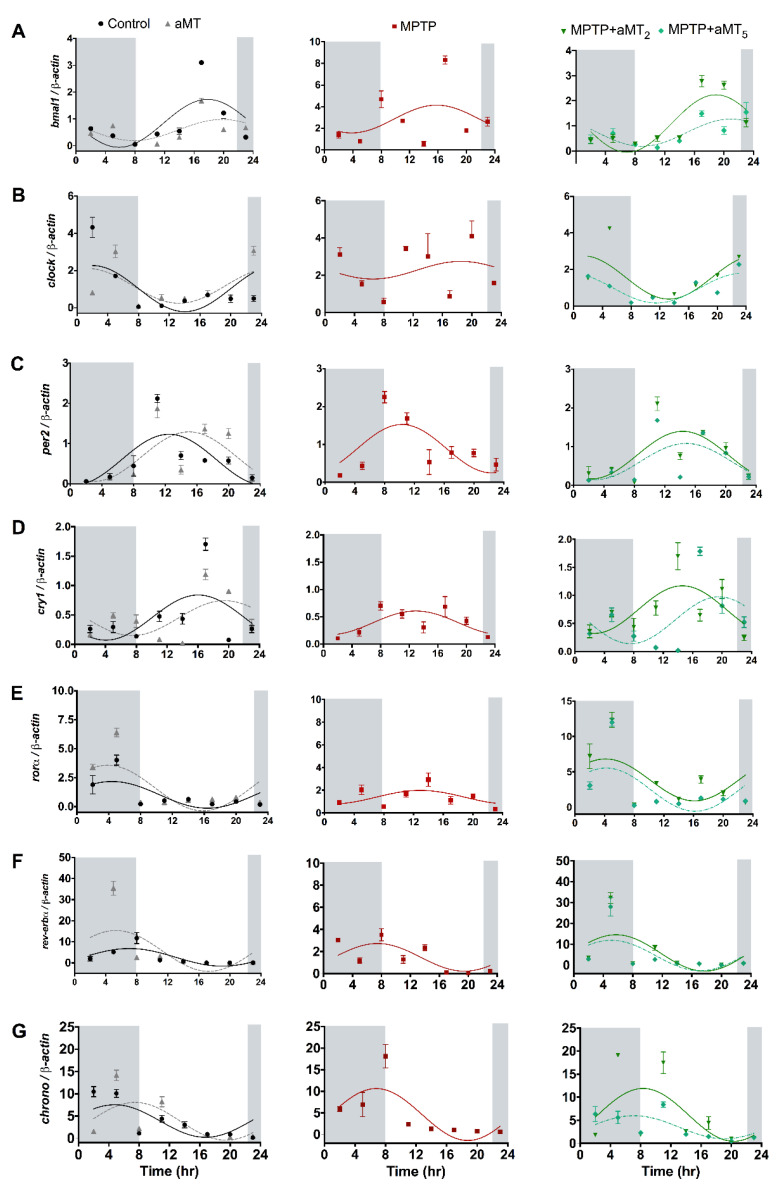
Relative expression of the clock genes: (**A**) *bmal1*, (**B**) *clock*, (**C**) *per2*, (**D**) *cry1*, (**E**) *ror*α, (**F**) *reverbα*, and (**G**) *chrono* in control, aMT, MPTP, and MPTP+aMT treatments of zebrafish embryos. Data are expressed as means ± SEM. Measured in light and dark conditions (shaded part).

Regarding regulatory genes, the control group shows a *bmal1* rhythm that was unmodified by aMT (Table S2.1) but blunted after MPTP administration (Figure 4A). Both types of melatonin treatments, aMT_2_ and aMT_5_, recovered normal *bmal1* rhythm (Figure 4A). There were no significant differences between both treatments of melatonin plus MPTP and the control and aMT groups (Table S2.1). 

The rhythm in the other regulator gene, *clock*, was also detected in the control group (Figure 4B) and it was also unaffected by aMT (Table S2.2). As with *bmal1*, the MPTP treatment lost the rhythm of *clock* (Figure 4B), reversing its values during the day (Table S2.2). Here, melatonin therapies restored the rhythm of *clock* to the values in the control and aMT groups (Figure 4B). 

The inhibitor genes, *per2* and *cry1*, also express rhythms. The control group shows significant *per2* rhythm (Figure 4C). Here, the aMT and, unlike preceding genes, the MPTP treatments maintained the rhythm, although the latter induced a significant gene expression in the morning (Table S2.3). Melatonin administration to MPTP-treated embryos normalized the rhythm (Table S2.3). 

A rhythm in the other inhibitor, *cry1*, was also present in the control group, and it was maintained after aMT or MPTP treatment (Figure 4D), although with some differences at certain time points (Table S2.4). Preventive and curative treatments with aMT reinforced these rhythms (Table S2.4). 

The modulator gene, *rorα*, displayed rhythm in the control embryos, which was unaffected after aMT treatment (Figure 4E and Table S2.5). MPTP dampens and reverses these rhythms resulting in their absence, but these were restored with both aMT therapies. 

The control embryos also had a significant rhythm of the other modulator gene, *rev-erb*α, that was maintained after aMT and MPTP treatments, although the latter diminished the expression of the gene (Figure 4F and Table S2.6). The rhythm of *rev-erb*α in the MPTP group was restored to the control value after the curative and preventative aMT treatments. 

Lastly, we observed the rhythm of the modulator *chrono* in the control embryos, which was minimally affected by the aMT and MPTP treatments (Figure 4G and Table S2.7). Both aMT treatments in the MPTP embryos maintained the normal rhythm of *chrono*.

### 3.4. Changes in the Acrophase of Clock Genes Rhythms in Zebrafish Embryos Due to MPTP and/or aMT Treatments

**Figure 5:**
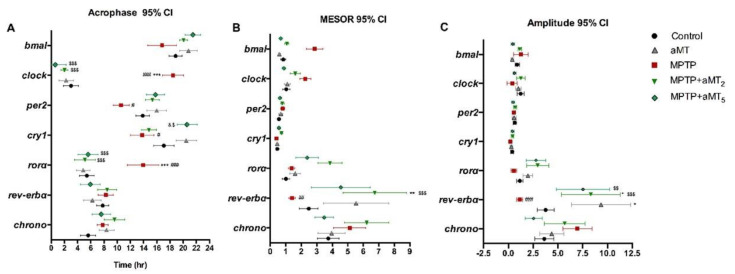
Graphical presentation of the cosinor parameters of the genes analyzed. (**A**) The acrophase, (**B**) Mesor, and (**C**) Amplitude chart of clock genes *bmal1*, *clock*, *per2*, *cry1*, *rorα*, *rev-erbα*, and *chrono*. Data are expressed as mean ± SEM. * *p* < 0.05 vs. control; ** *p* < 0.01 vs. control; *** *p* < 0.001 vs. control; # *p* < 0.05 vs. aMT; ## *p* < 0.01 vs. aMT; ### *p* < 0.001 vs. aMT; $ *p* < 0.05 vs. MPTP; $$ *p* < 0.01 vs. MPTP; $$$ *p* < 0.001 vs. MPTP; & *p* < 0.05 vs. MPTP+aMT2. Two-way ANOVA with a Tukey’s post hoc test.

Cosinor analysis of the acrophase of clock genes rhythms are depicted in the Supplementary Table S1, and are graphically presented in Figure 5A.

The acrophase in *bmal1* peaked at 19:00 h, and it was not modified significantly with aMT and was slightly delayed by MPTP, whereas aMT-primed embryos treated with MPTP had the same acrophase as the controls. The acrophase of *clock* occurred at 03:00 h in the control embryos and it was maintained after aMT treatment. MPTP treatment, however, induced a significant delay to 18:00 h that was restored to the control by aMT therapies. The control embryos displayed a *per2* acrophase at 14:00 h, and it was maintained after aMT, whereas the MPTP embryos underwent a non-significant advance of phase up to 10:00 h. The therapies with aMT in MPTP-treated embryos restored absolutely the acrophase of the rhythm. The acrophase of *cry1* occurred at 17:00 h in the controls and melatonin induced a non-significant phase advance up to 20:00. In this case, MPTP produced a phase delay peaking the rhythm at 14:00 h, which was restored to the controls after aMT therapies. Regarding *ror*α, its rhythm peaked at 06:00 h in the controls and in the aMT-treated embryos, advancing to 13:00 h after MPTP treatment. Both therapies with aMT reestablished the acrophase of the MPTP-treated embryos to the controls. The acrophase of *rev-erbα* occurred at 08:00 h, and it was slightly advanced at 06:00 h after aMT. The acrophase of *rev-erbα* was unmodified by any other treatment. Minimal changes in the acrophase occurred with the *chrono* rhythm among the different groups of treatments.

### 3.5. Changes in the Mesor of Clock Genes Rhythm in Zebrafish Embryos due to MPTP and/or aMT Treatments

Cosinor analysis of the MESOR of clock genes rhythms are depicted in Supplementary Table S1 and are graphically presented in Figure 5B. The control values of the MESOR of *bmal1* and *clock* were unmodified by aMT but increased after MPTP and were restored by aMT therapies to the controls (Figure 5B). In contrast, the *per2* and *cry1* MESOR were unchanged by any treatment (Figure 5B). We found that the MESOR of *ror*α was faintly higher in the MPTP groups treated with melatonin (Figure 5B). Moreover, the control and the MPTP group showed a decrease in the *rev-erbα* MESOR versus the melatonin treatments, significantly with respect to the aMT group (Figure 5B). Any treatment significantly affected the *chrono* MESOR (Figure 5B).

### 3.6. Changes in the Amplitude of Clock Genes Rhythm in Zebrafish Embryos Due to MPTP and/or aMT Treatments

Cosinor analysis of the amplitude of clock genes rhythms are depicted in Supplementary Table S1 and are graphically presented in Figure 5C. Similar results were found after analyzing the amplitude. MPTP treatment does not affect the amplitude of *bmal1*, *clock*, and *per2* (Figure 5C). As with MESOR, the *cry1* amplitude of the MPTP+aMT_2_ group was unchanged (Figure 5C). We also did not observe differences in the amplitude of *ror*α (Figure 5C). Nevertheless, *rev-erbα* presented a significantly lower acrophase in the control and the MPTP group versus aMT, in addition to the MPTP treatment versus its equivalent plus melatonin for 2 days (Figure 5C). Finally, the *chrono* amplitude did not have significant differences between any group (Figure 5C). 

### 3.7. Parkinsonian Zebrafish Had Impaired Mitochondrial Dynamics

**Figure 6:**
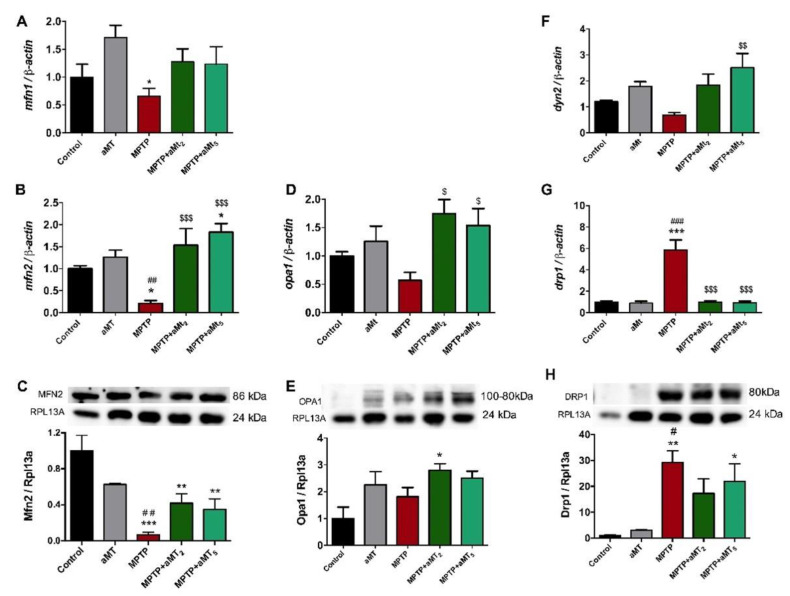
Analysis of the genes and proteins involved in mitochondrial dynamics. Regarding fission, the following markers were analyzed: (**A**) Expression of *mfn1* mRNA; (**B**) Expression of *mfn2* mRNA and (**C**) levels of Mfn2 protein in the same experimental groups; (**D**) Expression of *opa1* mRNA and (**E**) levels of Opa1 protein in the same experimental groups. Fusion markers analyzed were: (**F**) *dyn2* mRNA expression; (**G**) *drp1* mRNA expression; and (**H**) Drp1 protein levels in the same experimental groups. Data are presented as mean ± SEM. * *p* < 0.05 versus control; ** *p* < 0.01 vs. control; *** *p* < 0.001 vs. control; # *p* < 0.05 vs. aMT; ## *p* < 0.01 vs. aMT; ### *p* < 0.001 vs. aMT; $ *p* < 0.05; $$ *p* < 0.01 vs. MPTP; $$$ *p* < 0.001 vs. MPTP. One-way ANOVA with a Tukey’s post hoc test.

Because mitochondria is under the control of clock genes, and mitochondrial dysfunction is involved in the pathogenesis of PD [21,22], we analyzed the mechanisms of fusion and fission that reflect mitochondrial dynamics. Regarding fusion, we found that the expression of both mitofusins, *mfn1* and *mfn2*a, in the control embryos was unmodified by aMT treatment. Their expression, however, significantly decreased with MPTP administration (Figure 6A,B). The treatment with the two schedules of melatonin to the MPTP embryos recovered *mfn1* and, mainly, *mnf2* expression, the latter even above the control (Figure 6A,B). The protein levels of Mfn2 were also reduced by MPTP and recovered by aMT therapies (Figure 6C). We also observed a similar expression of *opa1* in the control and aMT-treated embryos that was reduced by MPTP. Here, both melatonin treatments to the MPTP embryos recovered the expression of *opa1* (Figure 6D). The protein levels of Opa1 were unmodified by MPTP or aMT, but they increased after aMT_2_ treatment (Figure 6E). In relation to mitochondrial fission, we found that the expression of *dyn2* mRNA was unmodified by aMT and was reduced by MPTP, an effect that was counteracted by the two melatonin treatments, aMT_2_ and aMT_5_ (Figure 6F). In turn, *drp1* mRNA expression and Drp1 protein content were unmodified by aMT treatment to the control embryos, but they increased significantly after MPTP. The protein and content mRNA expressions were significantly reduced with aMT therapies in the MPTP embryos (Figure 6G,H). 

### 3.8. MPTP Administration Altered Apoptosis and Autophagy Pathways

**Figure 7:**
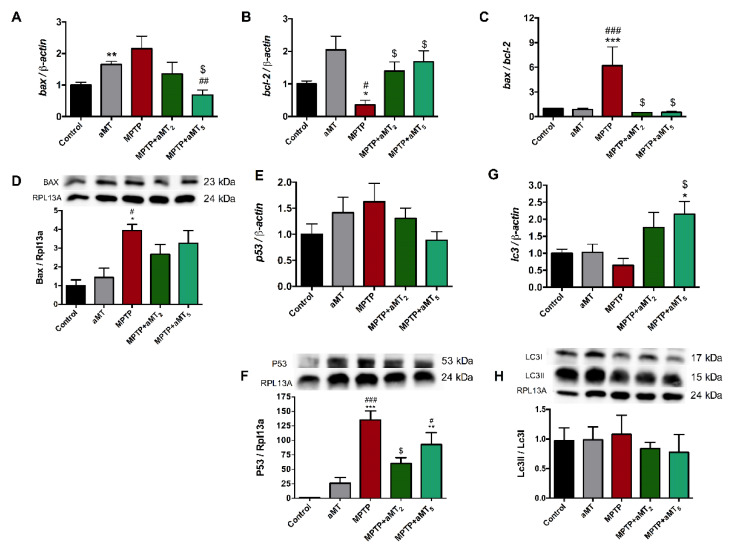
Analysis of apoptotic and autophagy pathways. (**A**) *bax* and (**B**) *bcl-2* mRNA expression in the studied groups, and the *bax/bcl-2* ratio (**C**). (**D**) protein levels of Bax. (**E**) p53 mRNA expression and (**F**) P53 protein levels. (**G**) mRNA expression of lc3 and (**H**) protein ratio of Lc3II/Lc3I. Data are presented as mean ± SEM. * *p* < 0.05 vs. control; ** *p* < 0.01 vs. control; *** *p* < 0.001 vs. control; # *p* < 0.05 vs. aMT; ## *p* < 0.01 vs. aMT; ### *p* < 0.001 vs. aMT; $ *p* < 0.05 vs. MPTP. Unpaired t test and One-way ANOVA with a Tukey’s post hoc test.

Mitochondrial dysfunction ultimately leads to autophagy and apoptosis of the dopaminergic neurons [23]. The mRNA expression of *bax* and the Bax protein content were slightly increased by aMT and MPTP, whereas aMT treatment, mainly aMT_5_, reduced it to the control (Figure 7A). *bcl-2*, in return, was unmodified by melatonin and was drastically reduced by MPTP; here, aMT_2_ and aMT_5_ restored its expression (Figure 7B). The ratio *bax/bcl-2* increased in parkinsonian embryos and was counteracted by both aMT treatments (Figure 7C). On the other hand, *p53* mRNA expression was unmodified by MPTP or melatonin treatments (Figure 7E). The protein content of P53, however, was significantly increased by MPTP and reduced after aMT_2_ and aMT_5_ treatments (Figure 7F). Our results did not show significant changes in *lc3* mRNA expression except for aMT_5_ treatment, and in the Lc3II/Lc3I ratio (Figure 7H). 

## 4. Discussion

Our results support for the first time that parkinsonism induced by MPTP in zebrafish embryos disturbs the normal working of the biological clock, altering the expression of main clock genes, including *bmal1*, *clock*, *per2*, *cry1*, *rorα*, *rev-erbα*, and *chrono*. 

It is known that disruption to the zebrafish clock affects circadian behavior [24] and motility [25], alters immunity [6] and the inflammatory response [26], affects the oxidative status [27], disturbs autophagy [28], and affects the mitochondrial function [29]. Thus, the chronodisruption here reported in parkinsonian zebrafish explains previous findings demonstrating the locomotor activity alteration, neuroinflammation, oxidative stress and dopaminergic neuronal loss, and mitochondrial disfunction in the same experimental model of the disease [16,17]. Here, we extend these findings showing how chronodisruption caused by MPTP results in day/night alteration in melatonin and motor activity rhythms, in mitochondrial dynamics, and in apoptotic/autophagic events whereas melatonin administration is able restore the normal rhythm of the clock genes, avoiding these clock genes-related misfunctions. 

Melatonin has significant neuroprotective properties in different models of PD including mouse and zebrafish [16,30] and, so, we first analyzed whether MPTP treatment may affect the endogenous levels of melatonin in parkinsonian embryos. As expected, melatonin levels show the typical day/night oscillation with higher levels during the dark phase. MPTP administration does not affect the day levels of melatonin but significantly blunts its nocturnal levels, blocking the daily rhythm of the indoleamine. Because the daily rhythm of dopamine in the striatum is controlled by melatonin [31], the alteration in the endogenous dopaminergic system in the brains of MPTP-treated zebrafish embryos elsewhere reported may be related to the lack of melatonin at night. Melatonin treatment to control and to MPTP-treated embryos does not only yield a significant increase in the day/night levels of melatonin but significantly increases them at night, reestablishing the circadian rhythm. The higher increase in melatonin at night, after its administration, compared with its levels during the daytime is not easy to explain, but changes in its adsorption or its catabolism could explain these findings, even though the embryos are exposed to exogenous melatonin for 24 h. Nevertheless, 24-h melatonin exposure is a common experimental condition able to restore cognitive and endocrine deficits in chronodisrupted zebrafish [32].

The alteration of melatonin levels is related to circadian rhythm disturbances, including the locomotor activity rhythm, which is lost in PD zebrafish [16]. Zebrafish, a nocturnal animal, are active during the day, showing a typical 24-h rhythm of motor activity that starts at lights on (08:00) [33]. This rhythm was affected by MPTP treatment, inducing a phase advance of 3–4 h, starting the activity at 5 am instead of 8 am. Probably, the advance occurs because of the alteration in melatonin production, since it controls the rhythm of activity in zebrafish [12]. Consequently, the embryos are more active during the night instead of the day. Motor alterations and incoordination after MPTP were also reported [34]. Melatonin counteracts this rhythm disorder but it reduces the total motor activity. Whereas the chrono synchronization properties of melatonin explains the rhythm normalization [35], the drop in motor activity may depend on the melatonin promotion of a sleepy state in zebrafish [36]. This effect of melatonin to restore motor activity has been observed in other models of neurodegeneration [37,38,39].

Hence, the alterations in the rhythm of melatonin and in motor activity in the embryos reported here point to an alteration in the circadian control of these activities, which may be caused by an alteration of the biological clock during the parkinsonian state. Therefore, we assessed a cosinor analysis of the expression of the clock genes during the 24-h period, to verify the rhythm. Our results support the normal rhythm of *bmal1*, *clock*, *per2*, *cry1, rorα*, and *rev-erb*α genes [6]. Parkinsonian embryos lost the rhythm of *bmal1*, *clock*, and *rorα*, which promoted an advance of *per2* acrophase, whereas this decreased the expression of *cry1* and *rev-erb*α genes. The rhythm of *chrono* was unaffected. There were differences in the MESOR but not in the amplitude of *bmal1* and *clock*, which increased in parkinsonian fish. In all cases, both melatonin treatments restored the normal rhythm of the clock genes. Overall, MPTP causes a parkinsonian condition in zebrafish embryos in parallel to a process of chronodisruption affecting most of the clock genes, which may account for the alteration in the rhythm of motor activity and probably melatonin production, but it may have other important consequences related with neuroinflammation, dopamine loss, and mitochondrial dysfunction. 

Firstly, clock genes modulate the innate immunity including the N-κB-dependent pathway and the production of proinflammatory cytokines such as Il-1β, Il-6, Tnfα, and Inf-γ [6,26] in such a form that chronodisruption switches to a proinflammatory state. The subsequent neuroinflammation [16] participates in the accumulation of γ-Synuclein in the case of zebrafish and activates the Nlrp3 inflammasome, the other pathway of the innate immunity, that exacerbates the production of proinflammatory cytokines and inhibits the activity of the antioxidant enzymes [40]. These cytokines inhibit the transcriptional activities of Bmal1/Clock, forming a feedback loop characteristic of PD [41]. 

Besides dopaminergic neuronal death [16], MPTP also promotes mitochondrial fragmentation in zebrafish [42], a finding that may be related to the alteration of clock genes and mitochondrial dynamics, as it occurs in other animal species [43,44]. Our results report a decrease in mitochondrial fusion through the inhibition of *mtf1* and *mtf2*, and an increase in mitochondrial fission indicated by the increase in *drp1* in embryos treated with MPTP. These changes in mitochondrial dynamics point to an increased mitophagy that collaborates in the neurodegeneration process [42]. Melatonin recovered normal mitochondrial dynamics activity, which is consistent with our data on mitochondrial function previously published in the same experimental model [17]. 

The circadian clock is also involved in the regulation of the antioxidant enzymes that are significantly affected in zebrafish PD [17,40,45,46]. Oxidative stress, together with the inflammation and mitochondrial damage mentioned above, leads to cell death and dopaminergic neurodegeneration. Here, we show an increased *bax*/*bcl-2* ratio as well as an increase in Bax and P53 proteins indicating increased apoptosis in MPTP-treated embryos, together with minor changes in autophagy. Treatments with melatonin reversed these changes to the control values. 

## 5. Conclusions

Together, we report here the alteration of the main clock genes in zebrafish embryos that have become parkinsonian with MPTP, which is accompanied by typical alterations in the rhythm and motor abilities of these embryos. Moreover, these embryos also display melatonin rhythm and mitochondrial failures, most of which are associated with the disturbance of clock genes. Melatonin administration, whose levels were reduced by MPTP, restored the normal functioning of the chronobiological clock, counteracting the parkinsonism.

## Figures and Tables

**Table 1:** Forward and reverse sequencies of the primers used for PCR.

**Gene**	**Accession Number**	**Forward Primer**	**Reverse Primer**
*bmal1*	NM_131577	AGGGCAGCACCACTGAATAC	TCCCTTCCCAGTGTCTTGTC
*clock*	NM_130957	GCAAACATCCAGAGACAGCA	ACTCGGCTGCAGAAACATCT
*per2*	NM_182857	AGGCTTTGGGGAAAGTCAAT	GCAGAATATGGCGTCTGGAT
*cry1*	NM_001077297	CACGCTCTACGATCTGGACA	GGAAGCGCTTATACGTGAGC
*ror* *α*	NM_001110167	TCGACCCTCAGAACAACACA	CCCAAACTCGAAGACAGAGC
*rev-erb* *α*	NM_200647	CCTCCCAAACACCTGAAAAA	CGCTGTTCATCTCTTGTCCA
*chrono*	NM_001327898	CGGTTGTGGAGGTAGCATTT	GTGCTACAATGGCCGACTTT
*mfn1*	NM_200647	AACGAAGTGTGCTCTGCTCA	GGATTCAGAGTTCGCCACCA
*mfn2*	NM_001128254	ACACATTTGCCACCTCTTCC	AGGCACGTGAGAGCCTAAAA
*opa1*	NM_001007298	AGACTGGAAGCAGAGGTGGA	GGAAGTGACGTCGAAAGAGC
*drp1*	NM_200922	AACATCCAGGACAGCGTACC	TCACCACAAGTGCGTCTCTC
*dyn2*	NM_200099	CGCAGATAGCAGTTGTCGGA	TCTGCTTCAATCTCCTGCCG
*bax*	NM_131562	AACTGGGGAAGAGTTGTGGC	GGGTGCCAAAATAACTGCGG
*bcl-2*	NM_001030253	CGAGTTTGGTGGGACCATGT	CGTACATCTCCACGAAGGCA
*p53*	NM_001271820	GAATCCCCAAAACTCCACGC	GGATGGCTGAGGCTGTTCTT
*lc3*	NM_199604	GGAGAGAAGCAACTGCCGAT	CCTGATTGGATGGGGAAGGG

## Data Availability

The data are contained within this article and the supplementary files.

## Supplementary Materials

The following supporting information can be downloaded at: https://www.mdpi.com/article/10.3390/antiox12040954/s1, Table S1: Cosinor analysis; Table S2.1–2.7: Post-hoc test: Tukey multiple comparison of the means of each clock gene.

## References

[1] Mantovani S., Smith S.S., Gordon R., O’sullivan J.D. (2018). An Overview of Sleep and Circadian Dysfunction in Parkinson’s Disease. J. Sleep Res..

[2] Hickey M.G., Demaerschalk B.M., Caselli R.J., Parish J.M., Wingerchuk D.M. (2007). “Idiopathic” Rapid-Eye-Movement (REM) Sleep Behavior Disorder Is Associated with Future Development of Neurodegenerative Diseases. Neurologist.

[3] Sowers J.R., Vlachakis N. (1984). Circadian Variation in Plasma Dopamine Levels in Man. J. Endocrinol. Investig..

[4] Bordet R., Devos D., Brique S., Touitou Y., Guieu J.D., Libersa C., Destée A. (2003). Study of Circadian Melatonin Secretion Pattern at Different Stages of Parkinson’s Disease. Clin. Neuropharmacol..

[5] Bonuccelli U., Del Dotto P., Lucetti C., Petrozzi L., Bernardini S., Gambaccini G., Rossi G., Piccini P. (2000). Diurnal Motor Variations to Repeated Doses of Levodopa in Parkinson’s Disease. Clin. Neuropharmacol..

[6] Sacksteder R.E., Kimmey J.M. (2022). Immunity, Infection, and the Zebrafish Clock. Infect. Immun..

[7] Cahill G.M. (2002). Clock Mechanisms in Zebrafish. Cell Tissue Res..

[8] Goriki A., Hatanaka F., Kim M.J., Yoritaka J.K. (2014). A Novel Protein, CHRONO, Functions as a Core Component of the Mammalian Circadian Clock. PLoS Biol..

[9] Anafi R.C., Lee Y., Sato T.K., Venkataraman A., Ramanathan C. (2014). Machine Learning Helps Identify CHRONO as a Circadian Clock Component. PLoS Biol..

[10] Hastings M.H., Maywood E.S., Brancaccio M. (2018). Generation of Circadian Rhythms in the Suprachiasmatic Nucleus. Nat. Rev. Neurosci..

[11] Lima-Cabello E., Díaz-Casado M.E., Guerrero J.A., Otalora B.B., Escames G., Lõpez L.C., Reiter R.J., Acuña-Castroviejo D. (2014). A Review of the Melatonin Functions in Zebrafish Physiology. J. Pineal Res..

[12] Aranda-martínez P., Fernández-martínez J., Ramírez-casas Y., Guerra-librero A., Rodríguez-santana C., Escames G., Acuña-castroviejo D. (2022). The Zebrafish, an Outstanding Model for Biomedical Research in the Field of Melatonin and Human Diseases. Int. J. Mol. Sci..

[13] Yang M., Huang J., Zhang S., Zhao X., Feng D., Feng X. (2021). Melatonin Mitigated Circadian Disruption and Cardiovascular Toxicity Caused by 6-Benzylaminopurine Exposure in Zebrafish. Ecotoxicol. Environ. Saf..

[14] Genario R., Giacomini A.C.V.V., Demin K.A., dos Santos B.E., Marchiori N.I., Volgin A.D., Bashirzade A., Amstislavskaya T.G., de Abreu M.S., Kalueff A.V. (2019). The Evolutionarily Conserved Role of Melatonin in CNS Disorders and Behavioral Regulation: Translational Lessons from Zebrafish. Neurosci. Biobehav. Rev..

[15] Acuña-Castroviejo D., Escames G., Leon L., Carazo A., Khaldy H. (2003). Mitochondrial regulation by melatonin and its metabolites. Adv. Exp. Med. Biol..

[16] Díaz-Casado M.E., Lima E., García J.A., Doerrier C., Aranda P., Sayed R.K.A., Guerra-Librero A., Escames G., López L.C., Acuña-Castroviejo D. (2016). Melatonin Rescues Zebrafish Embryos from the Parkinsonian Phenotype Restoring the Parkin/PINK1/DJ-1/MUL1 Network. J. Pineal Res..

[17] Díaz-Casado M.E., Rusanova I., Aranda P., Fernández-Ortiz M., Sayed R.K.A., Fernández-Gil B.I., Hidalgo-Gutiérrez A., Escames G., López L.C., Acuña-Castroviejo D. (2018). In Vivo Determination of Mitochondrial Respiration in 1-Methyl-4-Phenyl-1,2,3,6-Tetrahydropyridine-Treated Zebrafish Reveals the Efficacy of Melatonin in Restoring Mitochondrial Normalcy. Zebrafish.

[18] Westerfield M. (2007). The Zebrafish Book. A Guide for the Laboratory Use of Zebrafish (Danio Rerio).

[19] Videnovic A., Golombek D. (2017). Circadian Dysregulation in Parkinson’s Disease. Neurobiol. Sleep Circadian Rhythm..

[20] Moustafa A.A., Chakravarthy S., Phillips J.R., Gupta A., Keri S., Polner B., Frank M.J., Jahanshahi M. (2016). Motor Symptoms in Parkinson’s Disease: A Unified Framework. Neurosci. Biobehav. Rev..

[21] Phillipson O.T. (2017). Alpha-Synuclein, Epigenetics, Mitochondria, Metabolism, Calcium Traffic, & Circadian Dysfunction in Parkinson’s Disease. An Integrated Strategy for Management. Ageing Res. Rev..

[22] Scrima R., Cela O., Merla G., Augello B., Rubino R., Quarato G., Fugetto S., Menga M., Fuhr L., Relógio A. (2016). Clock-Genes and Mitochondrial Respiratory Activity: Evidence of a Reciprocal Interplay. Biochim. Et Biophys. Acta (BBA) Bioenerg..

[23] Wang B., Abraham N., Gao G., Yang Q. (2016). Dysregulation of Autophagy and Mitochondrial Function in Parkinson’s Disease. Transl. Neurodegener..

[24] Ben-Moshe Livne Z., Alon S., Vallone D., Bayleyen Y., Tovin A., Shainer I., Nisembaum L.G., Aviram I., Smadja-Storz S., Fuentes M. (2016). Genetically Blocking the Zebrafish Pineal Clock Affects Circadian Behavior. PLoS Genet..

[25] Basti A., Fior R., Yalçin M., Póvoa V., Astaburuaga R., Li Y., Naderi J., Godinho Ferreira M., Relógio A. (2020). The Core-Clock Gene NR1D1 Impacts Cell Motility In Vitro and Invasiveness in A Zebrafish Xenograft Colon Cancer Model. Cancers.

[26] Ren D., Zhang J., Yang L., Wang X., Wang Z., Huang D., Tian C., Hu B. (2018). Circadian Genes Period1b and Period2 Differentially Regulate Inflammatory Responses in Zebrafish. Fish Shellfish. Immunol..

[27] Alifu Y., Kofuji S., Sunaga S., Kusaba M., Hirayama J., Nishina H. (2021). The Light-Inducible Genes Per2, Cry1a, and Cry2a Regulate Oxidative Status in Zebrafish. Biol. Pharm. Bull..

[28] Huang G., Zhang F., Ye Q., Wang H. (2016). The Circadian Clock Regulates Autophagy Directly through the Nuclear Hormone Receptor Nr1d1/Rev-Erbα and Indirectly via Cebpb/(C/Ebpβ) in Zebrafish. Autophagy.

[29] Dao P., Hajny S., Mekis R., Orel L., Dinhopl N., Tessmar-Raible K., Nowikovsky K. (2022). The Cation Exchanger Letm1, Circadian Rhythms, and NAD(H) Levels Interconnect in Diurnal Zebrafish. Life Sci. Alliance.

[30] Khaldy H., Escames G., León J., Bikjdaouene L., Acuña-Castroviejo D. (2003). Synergistic Effects of Melatonin and Deprenyl against MPTP-Induced Mitochondrial Damage and DA Depletion. Neurobiol. Aging.

[31] Khaldy H., León J., Escames G., Bikjdaouene L., García J.J., Acuña-Castroviejo D. (2002). Circadian Rhythms of Dopamine and Dihydroxyphenyl Acetic Acid in the Mouse Striatum: Effects of Pinealectomy and of Melatonin Treatment. Neuroendocrinology.

[32] Giacomini A.C.V.V., Teixeira K.H., Marcon L., Scolari N., Bueno B.W., Genario R., de Abreu N.S., Demin K.A., Galstyan D.S., Kalueff A. (2020). Melatonin Treatment Reverses Cognitive and Endocrine Deficits Evoked by a 24-h Light Exposure in Adult Zebrafish. Neurosci. Lett..

[33] HURD M., DEBRUYNE J., STRAUME M., CAHILL G. (1998). Circadian Rhythms of Locomotor Activity in Zebrafish. Physiol. Behav..

[34] Razali K., Mohd Nasir M.H., Othman N., Doolaanea A.A., Kumar J., Nabeel Ibrahim W., Mohamed W.M.Y. (2022). Characterization of Neurobehavioral Pattern in a Zebrafish 1-Methyl-4-Phenyl-1,2,3,6-Tetrahydropyridine (MPTP)-Induced Model: A 96-Hour Behavioral Study. PLoS ONE.

[35] Acuña-Castroviejo D., Rahim I., Acuña-Fernández C., Fernández-Ortiz M., Solera-Marín J., Sayed R.K., Díaz-Casado M.E., Rusanova I., López L.C., Escames G. (2017). Melatonin, Clock Genes and Mitochondria in Sepsis. Cell. Mol. Life Sci..

[36] Zhdanova I.V., Wang S.Y., Leclair O.U., Danilova N.P. (2001). Melatonin Promotes Sleep-like State in Zebrafish. Brain Res..

[37] Zheng R., Ruan Y., Yan Y., Lin Z., Xue N., Yan Y., Tian J., Yin X., Pu J., Zhang B. (2021). Melatonin Attenuates Neuroinflammation by Down-Regulating NLRP3 Inflammasome via a SIRT1-Dependent Pathway in MPTP-Induced Models of Parkinson’s Disease. J. Inflamm. Res..

[38] Yildirim F.B., Ozsoy O., Tanriover G., Kaya Y., Ogut E., Gemici B., Dilmac S., Ozkan A., Agar A., Aslan M. (2014). Mechanism of the Beneficial Effect of Melatonin in Experimental Parkinson’s Disease. Neurochem. Int..

[39] Bavithra S., Selvakumar K., Sundareswaran L., Arunakaran J. (2017). Neuroprotective Effect of Melatonin Against PCBs Induced Behavioural, Molecular and Histological Changes in Cerebral Cortex of Adult Male Wistar Rats. Neurochem. Res..

[40] Dongjie S., Rajendran R.S., Xia Q., She G., Tu P., Zhang Y., Liu K. (2022). Neuroprotective Effects of Tongtian Oral Liquid, a Traditional Chinese Medicine in the Parkinson’s Disease-Induced Zebrafish Model. Biomed. Pharmacother..

[41] Li Y., Xia Y., Yin S., Wan F., Hu J., Kou L., Sun Y., Wu J., Zhou Q., Huang J. (2021). Targeting Microglial α-Synuclein/TLRs/NF-KappaB/NLRP3 Inflammasome Axis in Parkinson’s Disease. Front. Immunol..

[42] Kalyn M., Ekker M. (2021). Cerebroventricular Microinjections of MPTP on Adult Zebrafish Induces Dopaminergic Neuronal Death, Mitochondrial Fragmentation, and Sensorimotor Impairments. Front. Neurosci..

[43] Xu L., Lin J., Liu Y., Hua B., Cheng Q., Lin C., Yan Z., Wang Y., Sun N., Qian R. (2022). CLOCK Regulates Drp1 MRNA Stability and Mitochondrial Homeostasis by Interacting with PUF60. Cell Rep..

[44] Schmitt K., Grimm A., Dallmann R., Oettinghaus B., Restelli L.M., Witzig M., Ishihara N., Mihara K., Ripperger J.A., Albrecht U. (2018). Circadian Control of DRP1 Activity Regulates Mitochondrial Dynamics and Bioenergetics. Cell Metab..

[45] Kou L., Chi X., Sun Y., Han C., Wan F., Hu J., Yin S., Wu J., Li Y., Zhou Q. (2022). The Circadian Clock Protein Rev-Erbα Provides Neuroprotection and Attenuates Neuroinflammation against Parkinson’s Disease via the Microglial NLRP3 Inflammasome. J. Neuroinflammation.

[46] Kim J., Jang S., Choi M., Chung S., Choe Y., Choe H.K., Son G.H., Rhee K., Kim K. (2018). Abrogation of the Circadian Nuclear Receptor REV-ERBα Exacerbates 6-Hydroxydopamine-Induced Dopaminergic Neurodegeneration. Mol. Cells.

