# Supplementary material for: Chronodisruption and Loss of Melatonin Rhythm, Associated with Alterations in Daily Motor Activity and Mitochondrial Dynamics in Parkinsonian Zebrafish, Are Corrected by Melatonin Treatment
Source: Antioxidants (Basel). 2023 Apr 18;12(4):954. doi: 10.3390/antiox12040954 (PMC10136267; doi:10.3390/antiox12040954)
Supplement: Supplementary file 1 [file antioxidants-12-00954-s001.zip › antioxidants-2285275-supplementary.pdf]

Table S1. Cosinor analysis

Transcript	Group	p	PR	Acrophase (hr)	Mesor	Amplitude
bmal1	Control	0,0013	47,04%	18,87 (16,93-20,73)	0,835 (0,531-1,14)	0,892 (0,463-1,32)
	aMT	0,0129	33,95%	20,80 (18,20-23,47)	0,586 (0,409-0,762)	0,394 (0,145-0,643)
	MPTP	0,2129	13,70%	16,80 (12,53-21,13)	2,86 (1,83-3,89)	1,28 (-0,179-2,74)
	MPTP+aMT $_2$	0,0000	68,64%	20,07 (18,87-21,20)	1,07 (0,823-1,32)	1,15 (0,795-1,50)
	MPTP+aMT $_5$	0,0063	38,32%	21,47 (19,13-23,80)	0,676 (0,491-0,861)	0,454 (0,193-0,716)
clock	Control	0,0041	40,81%	3,01 (0,81-5,22)	1,03 (0,550-1,51)	1,24 (0,561-1,91)
	aMT	0,0033	42,04%	2,25 (0,11-4,40)	1,10 (0,722-1,49)	1,01 (0,473-1,55)
	MPTP	0,7323	2,92%	18,47 (15,33-21,60)	2,26 (1,56-2,96)	0,379 (-0,611-1,37)
	MPTP+aMT $_2$	0,0263	29,28%	2,03 (0,96-5,02)	1,61 (0,980-2,24)	1,26 (0,371-2,15)
	MPTP+aMT $_5$	0,0013	46,78%	0,62 (-2,55-1,31)	0,872 (0,663-1,08)	0,610 (0,315-0,906)
per2	Control	0,0018	45,08%	13,87 (11,87-15,87)	0,558 (0,331-0,786)	0,643 (0,321-0,965)
	aMT	0,0195	31,28%	16,00 (13,13-18,80)	0,677 (0,400-0,953)	0,581 (0,190-0,972)
	MPTP	0,0083	36,64%	10,60 (8,20-13,07)	0,801 (0,560-1,04)	0,571 (0,230-0,911)
	MPTP+aMT $_2$	0,0019	44,88%	15,33 (13,27-17,33)	0,780 (0,539-1,02)	0,680 (0,338-1,02)
	MPTP+aMT $_5$	0,0141	33,36%	15,80 (13,13-18,47)	0,616 (0,404-0,827)	0,467 (0,168-0,767)
cry1	Control	0,0256	29,46%	17,07 (14,07-20,00)	0,455 (0,265-0,646)	0,384 (0,114-0,653)
	aMT	0,0291	28,59%	20,47 (17,40-23,53)	0,446 (0,296-0,597)	0,298 (0,0841-0,511)
	MPTP	0,0493	24,92%	13,80 (10,33-17,27)	0,394 (0,279-0,508)	0,205 (0,0436-0,367)
	MPTP+aMT $_2$	0,0044	40,31%	14,80 (12,53-17,00)	0,720 (0,555-0,886)	0,423 (0,190-0,657)
	MPTP+aMT $_5$	0,0254	29,51%	20,60 (17,60-23,53)	0,556 (0,349-0,763)	0,418 (0,125-0,711)
ror α	Control	0,0042	40,63%	5,41 (3,19-7,60)	1,01 (0,568-1,46)	1,15 (0,520-1,79)
	aMT	0,0017	45,45%	4,85 (2,87-6,87)	1,60 (0,912-2,30)	1,97 (0,990-2,95)
	MPTP	0,1753	15,28%	13,93 (9,27-18,67)	1,38 (0,961-1,80)	0,555 (-0,0381-1,15)
	MPTP+aMT $_2$	0,0331	27,72%	5,11 (1,97-8,27)	3,85 (2,31-5,38)	2,96 (0,792- 5,14)
	MPTP+aMT $_5$	0,0254	29,51%	5,59 (2,53-8,67)	2,37 (0,945-3,79)	2,78 (0,830-4,74)
rev-erb α	Control	0,0005	51,14%	7,80 (6,05-9,53)	2,48 (1,30-3,66)	3,77 (2,10-5,44)
	aMT	0,0120	34,37%	6,25 (3,66-8,87)	5,53 (1,39-9,68)	9,35 (3,49-15,2)
	MPTP	0,0040	40,91%	8,27 (6,05-10,47)	1,40 (0,966-1,84)	1,14 (0,516-1,75)
	MPTP+aMT $_2$	0,0243	29,81%	8,47 (5,62-11,33)	6,73 (2,73-10,7)	8,32 (2,50-14,1)
	MPTP+aMT $_5$	0,0251	29,59%	5,93 (2,97-8,87)	4,55 (0,820-8,29)	7,54 (2,26-12,8)
chrono	Control	0,0029	42,65%	5,60 (3,39-7,80)	3,74 (2,36-5,13)	3,62 (1,72-5,53)
	aMT	0,0044	40,37%	8,40 (6,15-10,60)	3,95 (2,24-5,66)	4,38 (1,97-6,80)
	MPTP	0,0003	54,61%	7,80 (6,19-9,47)	5,12 (3,09-7,16)	6,96 (4,08-9,84)
	MPTP+aMT $_2$	0,0266	29,22%	9,60 (6,59-12,60)	6,22 (3,39-9,06)	5,68 (1,67-9,69)
	MPTP+aMT $_5$	0,0170	32,18%	7,60 (4,87-10,40)	3,48 (2,29-4,66)	2,55 (0,870-4,23)

Table S2.1. Transcript: *bmal1*. Post-hoc test: Tukey multiple comparison of the means

**Treatments**	**P value**							
	2h	5h	8h	11h	14h	17h	20h	23h
Control vs aMT	ns	ns	ns	ns	ns	ns	P < 0.001	ns
Control vs MPTP	ns	ns	P < 0.001	P < 0.001	ns	P < 0.001	ns	P < 0.001
Control vs MPTP+aMT$_2$	ns	ns	ns	ns	ns	ns	ns	ns
Control vs MPTP+aMT$_5$	ns	ns	ns	ns	ns	P < 0.05	P < 0.01	ns
aMT vs MPTP	ns	ns	P < 0.001	P < 0.001	ns	P < 0.001	ns	P < 0.01
aMT vs MPTP+aMT$_2$	ns	ns	ns	ns	ns	ns	P < 0.001	ns
aMT vs MPTP+aMT$_5$	ns	ns	ns	ns	ns	ns	ns	ns
MPTP vs MPTP+aMT$_2$	ns	ns	P < 0.001	P < 0.001	ns	P < 0.001	ns	P < 0.05
MPTP vs MPTP+aMT$_5$	ns	ns	P < 0.001	P < 0.001	ns	P < 0.001	ns	ns
MPTP+aMT$_2$ vs MPTP+aMT$_5$	ns	ns	ns	ns	ns	P < 0.05	P < 0.01	ns

Table S2.2. Transcript: *clock*. Post-hoc test: Tukey multiple comparison of the means

**Treatments**	**P value**							
	2h	5h	8h	11h	14h	17h	20h	23h
Control vs aMT	P < 0.001	ns	ns	ns	ns	ns	ns	P < 0.05
Control vs MPTP	ns	ns	ns	P < 0.01	P < 0.05	P < 0.001	P < 0.001	ns
Control vs MPTP+aMT$_2$	P < 0.05	P < 0.05	ns	ns	ns	ns	ns	ns
Control vs MPTP+aMT$_5$	P < 0.05	ns	ns	ns	ns	ns	ns	ns
aMT vs MPTP	P < 0.05	ns	ns	P < 0.01	P < 0.01	P < 0.001	P < 0.001	ns
aMT vs MPTP+aMT$_2$	ns	ns	ns	ns	ns	ns	ns	ns
aMT vs MPTP+aMT$_5$	ns	ns	ns	ns	ns	ns	ns	ns
MPTP vs MPTP+aMT$_2$	ns	P < 0.01	ns	P < 0.01	P < 0.05	P < 0.001	P < 0.05	ns
MPTP vs MPTP+aMT$_5$	ns	ns	ns	P < 0.01	P < 0.01	P < 0.001	P < 0.001	ns
MPTP+aMT$_2$ vs MPTP+aMT$_5$	ns	P < 0.001	ns	ns	ns	ns	ns	ns

Table S2.3. Transcript: *per2*. Post-hoc test: Tukey multiple comparison of the means

Treatments	P value							
	2h	5h	8h	11h	14h	17h	20h	23h
Control vs aMT	ns	ns	ns	ns	ns	P < 0.01	P < 0.01	ns
Control vs MPTP	ns	ns	P < 0.001	ns	ns	ns	ns	ns
Control vs MPTP+aMT2	ns	ns	ns	ns	ns	P < 0.01	ns	ns
Control vs MPTP+aMT5	ns	ns	ns	P < 0.05	ns	P < 0.01	ns	P < 0.001
aMT vs MPTP	ns	ns	P < 0.001	ns	ns	ns	ns	ns
aMT vs MPTP+aMT2	ns	ns	ns	ns	ns	ns	ns	ns
aMT vs MPTP+aMT5	ns	ns	ns	ns	ns	ns	P < 0.05	P < 0.001
MPTP vs MPTP+aMT2	ns	ns	P < 0.001	ns	ns	ns	ns	ns
MPTP vs MPTP+aMT5	ns	ns	P < 0.001	ns	ns	ns	ns	ns
MPTP+aMT2 vs MPTP+aMT5	ns	ns	ns	P < 0.05	ns	ns	ns	P < 0.01

Table S2.4. Transcript: *cry1*. Post-hoc test: Tukey multiple comparison of the means

Treatments	P value							
	2h	5h	8h	11h	14h	17h	20h	23h
Control vs aMT	ns	ns	ns	ns	ns	ns	ns	ns
Control vs MPTP	ns	ns	ns	ns	ns	ns	ns	ns
Control vs MPTP+aMT2	ns	ns	ns	ns	ns	ns	ns	P < 0.001
Control vs MPTP+aMT5	ns	ns	ns	ns	ns	ns	ns	ns
aMT vs MPTP	ns	ns	ns	ns	ns	ns	ns	ns
aMT vs MPTP+aMT2	ns	ns	ns	ns	ns	ns	ns	P < 0.001
aMT vs MPTP+aMT5	ns	ns	ns	ns	ns	ns	ns	ns
MPTP vs MPTP+aMT2	ns	ns	ns	ns	ns	ns	ns	P < 0.001
MPTP vs MPTP+aMT5	ns	ns	ns	ns	ns	ns	ns	ns
MPTP+aMT2 vs MPTP+aMT5	ns	ns	ns	ns	ns	ns	ns	P < 0.001

Table S2.5. Transcript: *rorα*. Post-hoc test: Tukey multiple comparison of the means

**Treatments**	**P value**							
	2h	5h	8h	11h	14h	17h	20h	23h
Control vs aMT	ns	P < 0.001	ns	ns	ns	ns	ns	ns
Control vs MPTP	ns	ns	ns	ns	P < 0.001	ns	ns	ns
Control vs MPTP+aMT$_2$	P < 0.001	P < 0.001	ns	P < 0.001	ns	P < 0.001	ns	ns
Control vs MPTP+aMT$_5$	ns	P < 0.001	ns	ns	ns	ns	ns	ns
aMT vs MPTP	ns	P < 0.001	ns	ns	P < 0.01	ns	ns	ns
aMT vs MPTP+aMT$_2$	P < 0.001	P < 0.001	ns	P < 0.001	ns	P < 0.001	ns	ns
aMT vs MPTP+aMT$_5$	ns	P < 0.001	ns	ns	ns	ns	ns	ns
MPTP vs MPTP+aMT$_2$	P < 0.001	P < 0.001	ns	P < 0.05	P < 0.05	P < 0.001	ns	ns
MPTP vs MPTP+aMT$_5$	ns	P < 0.001	ns	ns	P < 0.001	ns	ns	ns
MPTP+aMT$_2$ vs MPTP+aMT$_5$	P < 0.001	ns	ns	P < 0.001	ns	P < 0.001	ns	ns

Table S2.6. Transcript: *rev-erbα*. Post-hoc test: Tukey multiple comparison of the means

**Treatments**	**P value**							
	2h	5h	8h	11h	14h	17h	20h	23h
Control vs aMT	ns	P < 0.001	P < 0.001	ns	ns	ns	ns	ns
Control vs MPTP	ns	ns	P < 0.001	ns	ns	ns	ns	ns
Control vs MPTP+aMT$_2$	ns	P < 0.001	P < 0.001	P < 0.001	ns	ns	ns	ns
Control vs MPTP+aMT$_5$	ns	P < 0.001	P < 0.001	ns	ns	ns	ns	ns
aMT vs MPTP	ns	P < 0.001	ns	ns	ns	ns	ns	ns
aMT vs MPTP+aMT$_2$	ns	P < 0.001	ns	P < 0.01	ns	ns	ns	ns
aMT vs MPTP+aMT$_5$	ns	P < 0.001	ns	ns	ns	ns	ns	ns
MPTP vs MPTP+aMT$_2$	ns	P < 0.001	ns	P < 0.001	ns	ns	ns	ns
MPTP vs MPTP+aMT$_5$	ns	P < 0.001	ns	ns	ns	ns	ns	ns
MPTP+aMT$_2$ vs MPTP+aMT$_5$	ns	ns	ns	P < 0.01	ns	ns	ns	ns

Table S2.7. Transcript: *chrono*. Post-hoc test: Tukey multiple comparison of the means

Treatments	P value							
	2h	5h	8h	11h	14h	17h	20h	23h
Control vs aMT	P < 0.01	ns	P < 0.001	ns	ns	ns	ns	ns
Control vs MPTP	P < 0.05	P < 0.05	P < 0.001	ns	ns	ns	ns	P < 0.05
Control vs MPTP+aMT_2	P < 0.01	P < 0.01	P < 0.001	P < 0.001	ns	ns	ns	ns
Control vs MPTP+aMT_5	ns	ns	P < 0.001	ns	ns	ns	ns	ns
aMT vs MPTP	P < 0.001	P < 0.001	ns	P < 0.05	ns	ns	ns	P < 0.05
aMT vs MPTP+aMT_2	ns	ns	ns	P < 0.001	ns	ns	ns	ns
aMT vs MPTP+aMT_5	ns	P < 0.01	ns	ns	ns	ns	ns	ns
MPTP vs MPTP+aMT_2	P < 0.001	P < 0.001	ns	P < 0.001	ns	ns	ns	ns
MPTP vs MPTP+aMT_5	P < 0.001	ns	ns	P < 0.01	ns	ns	ns	ns
MPTP+aMT_2 vs MPTP+aMT_5	ns	P < 0.001	ns	P < 0.001	ns	ns	ns	ns
